# Challenges and Pitfalls: Performing Clinical Trials in Patients With Congenital Diaphragmatic Hernia

**DOI:** 10.3389/fped.2022.852843

**Published:** 2022-04-15

**Authors:** Suzan Cochius - den Otter, Jan A. Deprest, Laurent Storme, Anne Greenough, Dick Tibboel

**Affiliations:** ^1^Intensive Care and Department of Pediatric Surgery, Erasmus MC University Medical Center, Rotterdam, Netherlands; ^2^Department of Obstetrics and Gynaecology, University Hospitals KU Leuven, Leuven, Belgium; ^3^Academic Department of Development and Regeneration, Biomedical Sciences, KU Leuven, Leuven, Belgium; ^4^Institute for Women's Health, University College London, London, United Kingdom; ^5^Metrics-Perinatal Environment and Health, University of Lille, Lille, France; ^6^Department of Neonatology, Jeanne de Flandre Hospital, Centre Hospitalier Universitaire de Lille, Lille, France; ^7^Center of Rare Disease Congenital Diaphragmatic Hernia, Jeanne de Flandre Hospital, Centre Hospitalier Universitaire de Lille, Lille, France; ^8^Neonatal Intensive Care Centre, King's College Hospital NHS Foundation Trust, London, United Kingdom; ^9^Department of Women and Children's Health, School of Life Course Sciences, Faculty of Life Sciences and Medicine, King's College London, London, United Kingdom; ^10^The Asthma UK Centre for Allergic Mechanisms in Asthma, King's College London, London, United Kingdom; ^11^NIHR Biomedical Centre, Guy's and St Thomas NHS Foundation Trust and King's College London, London, United Kingdom

**Keywords:** congenital diaphragmatic hernia, clinical trials, congenital anomaly, prenatal therapy, postnatal therapy

## Abstract

Congenital diaphragmatic hernia (CDH) is a rare developmental defect of the lungs and diaphragm, with substantial morbidity and mortality. Although internationally established treatment guidelines have been developed, most recommendations are still expert opinions. Trials in patients with CDH, more in particular randomized controlled trials, are rare. Only three multicenter trials in patients with CDH have been completed, which focused on fetoscopic tracheal occlusion and ventilation mode. Another four are currently recruiting, two with a focus on perinatal transition and two on the treatment of pulmonary hypertension. Herein, we discuss major challenges and pitfalls when performing a clinical trial in infants with CDH. It is essential to select the correct intervention and dose, select the appropriate population of CDH patients, and also define a relevant endpoint that allows a realistic duration and sample size. New statistical approaches might increase the feasibility of randomized controlled trials in patients with CDH. One should also timely perform the trial when there is still equipoise. But above all, awareness of policymakers for the relevance of investigator-initiated trials is essential for future clinical research in this rare disease.

## Introduction

Congenital diaphragmatic hernia (CDH) is a rare developmental defect of the lungs and diaphragm that occurs in 1 per 4,000–4,500 live births (1). Infants with CDH are born with a variable amount of lung hypoplasia and abnormal pulmonary vasculature, causing ventilatory insufficiency and pulmonary hypertension (PH). Nowadays, with the introduction of standardized care, survival is ~73% in well-established centers of expertise (2). Although these centers use internationally established treatment guidelines, most recommendations are still expert opinions (3, 4). Trials in patients with CDH, more in particular randomized controlled trials (RCTs), are rare (2, 5–10). Sometimes, patients with CDH are included in RCTs, with a subanalysis for the patients with CDH, although the trial may not be powered to be informative for the latter (11, 12). More often, CDH is an exclusion criterion for participation in an RCT (13, 14). When trials are successfully completed, extrapolation of the results to clinical practice becomes a matter of debate (15). This way, relevant research questions stay unanswered, or their conclusions remain questioned and hence are not implemented.

## Trials in Congenital Diaphragmatic Hernia

Over time, the focus of therapy and thus the research questions in CDH have changed. Until the 1980s, CDH was considered a surgical emergency. Thereafter, preoperative stabilization became mainstream, focusing on correcting acidosis and hypoxia (16). The use of aggressive ventilation strategies, however, in the hypoplastic lung caused barotrauma and a high incidence of pneumothorax. Wung et al. (17) reported a respiratory strategy to minimize the risk of iatrogenic lung injury and exacerbation of PH, which focused on the prevention of hyperventilation and hyperinflation. Since then, this strategy has been adopted worldwide, and surgical closure has changed into an elective procedure in “stable” patients. In 2010, the first postnatal management guidelines of the CDH EURO Consortium were published, initiating standardized care throughout Europe (18). Together with the introduction of these guidelines, the VICI trial started the first postnatal RCT exclusively in CDH patients. In this CDH Euro Consortium trial, conventional ventilation and high-frequency oscillatory ventilation were compared (19). The primary outcome was chronic lung disease and/or mortality on day 28. Unfortunately, the calculated number of 400 patients was never included. The study was concluded after the recruitment of 171 patients over a study period of 5 years, because of lower-than-anticipated inclusion rate, logistic issues with recruitment in different centers, lack of financial resources, and increasing fear for trial fatigue. The study showed no significant difference between ventilation modes but was underpowered to make this a firm conclusion. Other outcome parameters, including treatment failure, ventilation time, duration of inotropic support, and need for extracorporeal membrane oxygenation (ECMO), all showed a trend toward a more favorable outcome in the conventional ventilation group 2 (2). In parallel, prenatal interventions were developed, based on the assumption that the diagnosis can be made prenatally, and the severity can be assessed. These were initially based on the anatomical repair by open fetal surgery but later focused on the stimulation of lung development [reviewed in (20)]. The latter can be achieved by temporary tracheal occlusion, *via* a percutaneous approach. The first, single-center, RCT assessing improvement in survival following tracheal occlusion using a variety of techniques in fetuses with severe and moderate hypoplasia was finalized early because of a higher-than-expected survival in the postnatal management group and a high prematurity rate in the intervention group (10). The European centers that designed the percutaneous fetoscopic occlusion technique with a balloon [fetoscopic endoluminal tracheal occlusion (FETO)] (21), which is maternally more acceptable, moved from a phase I trial to a large cohort study (22, 23). In view of the apparently higher survival rates compared to historical controls (24) but the lack of evidence, they initiated the Tracheal Occlusion To Accelerate Lung growth (TOTAL) randomized clinical trials. The trials were performed in fetuses with severe and moderate hypoplasia born in expert fetoscopy centers that also used the standardized neonatal management protocol as defined by the CDH Euro consortium (3). In severe left-sided CDH, FETO performed between 27 and 29 weeks significantly improved survival at discharge from the neonatal intensive care unit [relative risk (RR): 2.67 (95% CI: 1.22–6.11)], however, with increased risk of prematurity [RR: 2.59 (1.59–4.52)] (5). In patients with moderate CDH, FETO performed between 30 and 32 weeks did not improve survival [RR: 1.27 (0.99–1.63)] or need for oxygen at 6 months of age, at increased risk of prematurity [RR: 2.86 (1.94–4.34)] (6). In a pooled analysis of the data, the overall survival following FETO is increased [RR: 1.78 (1.05–3.01)], and it seems that the difference between both trials may be due to the difference in the time point of balloon insertion (25). In retrospect, there was preclinical and some observational clinical evidence that earlier occlusion yields a more vigorous lung response, but at the same time, it increases prematurity risk; hence, it was debated as being a good strategy (26, 27). The risk of tracheomalacia secondary to tracheal occlusion was low in both trials, with an incidence of 1.9% (5, 6, 28).

While the hypoplastic lung is still a relevant problem, nowadays, the focus has shifted somewhat from the lung toward PH, which remains an important determinant of mortality and morbidity (29). There are currently two trials recruiting that focus on physiological-based cord clamping: the PinC trial (NCT04373902) and the CHIC trial (9). Neonatal resuscitation of infants with CDH remains highly challenging because of the failure of cardiorespiratory adaptation at birth. The baby is frequently cyanotic and bradycardic as soon as the umbilical cord is clamped. Traditionally, the umbilical cord is clamped and cut immediately after birth. Following cord clamping, umbilical venous return is lost, and left ventricular output becomes dependent on pulmonary blood flow. However, in CDH infants, an increase in pulmonary blood flow is delayed because of high pulmonary vascular resistance. Delaying cord clamping while the resuscitation maneuvers are started may (1) facilitate blood transfer from the placenta to the baby to augment circulatory blood volume; (2) avoid the loss of venous return and decrease in left ventricle filling caused by immediate cord clamping; and (3) prevent initial hypoxemia because of sustained uteroplacental gas exchange after birth when the cord is intact. The PINC trial, performed in Europe, focuses on decreasing the incidence of PH, defined as 2 out of the 4 following criteria: (1) right ventricular systolic pressure (RVSP) ≥2/3 systemic systolic pressure, (2) right ventricle (RV) dilatation/septal displacement or RV dysfunction +/– left ventricle dysfunction, (3) pre–post ductal SpO_2_ difference >10%, and (4) oxygenation index (OI) >20. In this trial, a standardized echocardiogram is implemented. In the CHIC trial conducted in the French Rare Disease Network, the aim is to investigate the efficacy of intact cord resuscitation on cardiorespiratory adaptation directly after birth by comparing APGAR score (9).

In parallel, two RCTs are recruiting CDH neonates in the search for the best initial therapy for PH (7, 8). In the CoDiNOS trial (7), again initiated within the CDH Euro Consortium, intravenous sildenafil is compared with iNO as initial therapy for PH in CDH patients. In this trial, PH is strictly defined, using the same criteria as in the PinC trial. Structural and standardized echocardiograms are performed at set times, with the additional aim of increasing the knowledge of PH and cardiac function in CDH patients. In the Milrinone in CDH trial, a trial performed within the Neonatal Research Network in the United States, milrinone is compared with placebo in CDH patients with mild-to-moderate PH, defined as an OI of 10 or higher (Table 1) (8).

**Table 1:** Randomized controlled trials in CDH.

**RCT**	**Started in**	**Intervention**	**Primary outcome**
Fetal tracheal occlusion (10)^*^	1999–2001	FETO vs. standard prenatal care for moderate to severe CDH	Survival at age of 90 days
VICI trial (2)	2008–2013	Conventional ventilation vs. high-frequency ventilation	Death until discharge or CLD on day 28
TOTAL trial (6)	2008–2019	FETO vs. standard prenatal care for moderate CDH	Infant survival until discharge from intensive care and survival without oxygen at 6 months
TOTAL trial (5)	2011–2020	FETO vs. standard prenatal care for severe CDH	Infant survival until discharge from intensive care
Milrinone in CDH (8)	2016	Milrinone vs. placebo	Change in OI after 24 h
CDH Optimisation of Neonatal Ventilation^*^	2016	Ventilation with different tidal volumes	Change in pressure time product of the diaphragm
CoDiNOS (7)	2017	Sildenafil vs. iNO	Change in OI after 12 h
PinC	2020	Physiological-based cord clamping vs. direct cord clamping	Incidence of PH in the first day of life
CHIC (9)	2020	Physiological-based cord clamping vs. immediate cord clamping	Apgar score at 1 and 5 min
HFO vs. HFJ ventilation^*^	2021	High-frequency oscillatory ventilation vs. high-frequency jet ventilation	OI at 24 h

*CDH, congenital diaphragmatic hernia; CLD, chronic lung disease; FETO, fetoscopic endoluminal tracheal occlusion; PH, pulmonary hypertension; iNO, inhaled nitric oxide; OI, oxygenation index; HFO, high-frequency oscillation; HFJ, high-frequency jet ventilation; RCT, randomized controlled trial.*

* *Single-center trial*.

## Challenges When Performing Trials in Patients With Congenital Diaphragmatic Hernia

### The Right Intervention at the Right Time for the Right Patient

When performing a trial in patients with CDH, either prenatal or postnatal using a pharmacological intervention, it is essential to first establish an adequate dosing regimen before evaluating efficacy. Although pharmacokinetic drug testing in adults is very common, in infants, dosing regimens are often an extrapolation from adult data, only corrected for body size (30). This assumes that fractioning of the dose will lead to similar plasma drug levels, hence assuming that children have similar renal, gastrointestinal, and hepatic functions as well as body composition as adults. This can result in over- or under-dosing, consequently leading to toxicity or reduced efficacy (31). For sildenafil, a pharmacokinetic model in infants with CDH was built, using a NONMEM approach, before starting a trial (32). With a loading dose of 0.4 mg/kg in 3 h followed by a continuous infusion of 1.6 mg/kg/day, adequate sildenafil plasma levels were achieved, 190 μg/L after the loading dose. The numbers, however, were too low to detect any correlation between these concentrations and the OI. Earlier, Steinhorn et al. tested this dosing regimen in a dose-escalation trial in infants with persistent PH of the newborn (PPHN), defined as signs of PH on echocardiography, an OI > 15, and no other anomalies (33). Again, numbers were too low to detect a strong correlation between the different dosing regimens, plasma concentrations, and clinical effects. But patients with a plasma concentration over 58 μg/L after the loading dose seemed to have a decreased OI 4 h later. Recently, Pierce et al. reported in the same population, newborns with PPHN and no other anomalies, no significant additional effect of sildenafil to iNO in the treatment of PPHN in an RCT (34). The dosing regimen was the second-lowest regimen that improved the OI in the study by Steinhorn et al. (33). Improvement, however, was observed in a combined set of, mostly higher, dosing regimens. Steady-state concentrations of this combined group were 123 ng/ml, but only 73 μg/ml in the group using this lower sildenafil dosing regimen. In the recent trial of Pierce et al. the steady-state concentration was only 52 μg/ml (34). One can assume that the plasma concentration should at least be 123 ng/ml in order to observe clinical effects, underlining the necessity of pharmacokinetic modeling. So the question remains whether sildenafil has an additional effect in patients with PPHN who are already treated with iNO and whether sildenafil was dosed appropriately in the trial by Pierce et al. Samples collected during the CoDiNOS trial will provide more insight into the dose–response correlation of sildenafil in CDH patients as well as its other pharmacodynamic effects.

But is sildenafil the right drug? Although sildenafil could play a role in the treatment of PH in CDH, one could argue that, from a pathophysiological standpoint, it would be more logical to investigate drugs that act on different pathways, instead of comparing drugs that act on the nitric oxide–cGMP pathway such as sildenafil and iNO. Furthermore, no alteration of the nitric oxide–cGMP pathway in CDH patients has been found, decreasing the chance that drugs acting on this pathway will be effective (35). In a clinical retrospective trial, sildenafil seems beneficial in less than half of the patients with CDH (36). Drugs that affect the endothelin pathway, such as bosentan, might be more successful. An increase in endothelin A and B receptor expression and ECE-1 enzyme is found in patients with CDH. This enzyme is responsible for the conversion of endothelin-1 to its active form (35). CDH patients with PH have higher endothelin-1 plasma levels than CDH patients without PH (37). But endothelin receptor antagonists are still only available in oral form, making them unsuitable for the treatment of postnatal PH in CDH patients before surgical correction. The third pathway involved in PH, the prostacyclin pathway, seems to be altered in CDH patients too, with a decrease of prostaglandin-I2 receptor expression. This could explain the negative effect of prostacyclin derivates on PH in CDH patients, although results are conflicting (35, 38, 39). To decrease the incidence of PH, sildenafil has been discredited for its use prenatally, even though preclinical data in animals with CDH are promising. The Dutch STRIDER study, a trial investigating the effect of sildenafil on fetal growth restriction unrelated to CDH, was suspended because of an increased incidence of PPHN and neonatal mortality (40). It is, however, questionable if these negative findings should be extrapolated to other conditions. Antenatal administered sildenafil reduces vascular branching in healthy fetal rabbits but decreases the incidence of PH in animals with CDH by increasing the number of peripheral vessels (41). A phase I–IIb was set up to evaluate *in vivo* transplacental passage of sildenafil in humans and specifically in infants with CDH (42–44). But despite the preclinical differences, this trial had to be halted unduly after the publication of the results of the STRIDER trial.

PH in CDH is often resistant to pulmonary vasodilators such as iNO. This is possibly due to coexisting right and left ventricular dysfunction (36, 45). Milrinone has both inotropic and lusitropic properties and potentially decreases pulmonary vascular resistance (46). In the trial currently recruiting, infants with CDH and an OI of >10 are randomized for milrinone or placebo. The primary outcome is a change in OI over 24 h. In 2011 and 2012, only 17% of infants born in centers within the NRN, a well-established US research network, received milrinone (8). But currently, it is often common practice, and this could decrease the willingness within the NRN to participate in the trial, decreasing recruitment rates.

The CoDiNOS trial is also suffering from recruiting issues, and this is partly caused by lower-than-anticipated recruitment due to strict inclusion criteria. Although an echocardiogram is often believed to be the best diagnostic tool in newborns, the incidence of PH on echocardiogram on day 1 of life overestimates the incidence of clinically relevant PH. High pulmonary pressures at that time are still part of the physiological transition. Only infants with clinically relevant PH, defined as PH on echocardiogram and clinical signs of PH, are included. This definition decreases the eligible population from 60% to around 30%. Although this negatively affects the inclusion rate, including mild cases dilutes the effect of an intervention. Moreover, the harm of intervention for these mild cases should be taken into account, although the side effects of sildenafil seem to be mild (47). The same problem applies to prenatal and perinatal interventions. Even though ultrasound and MRI have made it possible to identify the severity of lung hypoplasia in infants with CDH, it is still very difficult to predict the severity of PH, due to the difference in pre- and postnatal physiology (48). Better prenatal diagnostic techniques should be able to identify the fetuses and newborns at risk and predict who would benefit from entry in a clinical trial. This would improve the safety and efficacy of a trial. A core outcome set with strictly defined and relevant outcome parameters is currently under development for perinatal interventions (49). A core outcome set for postnatal interventions and long-term outcomes would help to be able to compare postnatal trials and their outcome.

### Is Congenital Diaphragmatic Hernia, a Heterogeneous Orphan Disease, a Condition That Is Amenable for a Trial Anyway?

But is an RCT as we know it in its present form, in heterogeneous orphan diseases such as CDH, the only or optimal tool to collect evidence-based information (50–53)? The VICI trial had recruitment problems and lacked adequate financial support. Also, the TOTAL trials were not financially supported apart from the setup of the database. This seriously affected the research infrastructure in participating centers. Currently, lack of financial support has a serious impact on the CoDiNOS trial. Other important limiting factors for recruitment are the delays caused by national drug authorities' approval in participating countries and problems with legislation and insurance. Many centers were so far unable to join the CoDiNOS trial, although the primarily responsible physicians did see the relevance of participating in such a trial. Performing an RCT in pediatric and neonatal critical care is challenging, especially when a high number of centers are needed due to the rarity of the condition or eligible study participants. Collaborating in a research network, such as the CDH EURO Consortium, increases the chance of success. Members of the consortium are often collaborating as one team with a common goal, helping each other to overcome local and national barriers (54). The regulatory framework conduct (Randomized) Clinical Trials in pregnant women and children, especially drug interventions or new medical devices, are increasingly stringent and differ between countries. For example, many countries and healthcare institutions insist researchers use a Clinical Trial Organization or to perform external safety audits, but these organizations and audits are often very expensive, absorbing a major part of the already limited budget of investigator-initiated trials. Interestingly enough, investigator-initiated research is significantly more frequently cited than industry-led trials (55). This demonstrates that investigator-initiated research is essential and has an impact on clinical practice, as data are generated in a real-world setting. Legislation should adjust to facilitate such trials instead of being obstructive, often without any proven benefit or added safety. This was acknowledged in the revision of the Directive of the European Commission in 2014, “getting better legislation in place soon is crucial to enable and encourage life-saving research,” but this did not result in a substantial practice change (56). In January 2022, the new European Clinical Trial Regulation will be implemented, to simplify and accelerate clinical trials within the European Union. By centrally submitting the study protocols for the European Union and synchronizing the leap time for the different medical ethics review processes, study centers in different countries can start recruiting subjects at the same time (57). With this regulation, conducting trials will hopefully become less complex within Europe. But in the TOTAL trial, centers from outside the European Union participated, and also in the CoDiNOS trial, centers from the United Kingdom, Australia, and Canada unsuccessfully intended to join. Worldwide research networks using a uniform approach concerning protocols and outcome measures as well as getting the regulatory bodies to cooperate and agree on uniformity in research procedures would improve the research climate for rare diseases such as CDH tremendously.

As to CDH, which is a rare disease, one would hope that the European Reference Networks, launched in 2017, would facilitate clinical research, which was amongst others one of the goals of ERNs. One of these networks is the European Reference Network for rare Inherited and Congenital (digestive and gastrointestinal) Anomalies (ERNICA). The CDH EURO Consortium, which has been existing longer and has a proven track record, is now affiliated with ERNICA, which may help to increase funding and resources. ERNs include patient organizations, but the latter did not wait and have been and still are involved in the funding and development of investigator-initiated trials. Their participation increases the clinical relevance of trials. For instance, the CoDiNOS trial is funded for an important part by CDH-UK, the CDH patient organization in the United Kingdom.

Another factor is the heterogeneous severity of the condition (from very mild to very severe). A large number of patients as well as stratification based on prenatal markers of severity are required to show statistical differences. For instance, for the CHIC trial, an estimated 600 infants are needed to demonstrate a difference in mortality. That is unrealistic, and thus proxies are being used as the primary outcome. It is likely that physiological-based cord clamping will become standard of care if the PINC or CHIC shows a statistical difference in primary endpoints, even without evidence of a decrease in mortality rate. The same applies to the CoDiNOS trial, in which the initial primary endpoint (incidence of PH at the age of 2 weeks) was changed to change in OI at 12 h. That lowered the patients needed from 330 to 90, without decreasing the relevance of the trial. In many neonatal trials on PPHN, OI is used as primary outcome (13, 14). Not only the severity of the condition is heterogeneous, but also the outcome between centers differs, and centers that treat a low number of patients have a worse outcome than the high-volume centers with both complex neonatal intensive care facilities and expertise in neonatal surgery (58). A benefit of performing trials within, for instance, the CDH Euro Consortium, is that the affiliated centers are expert high-volume centers that offer standardized care, improving the baseline outcome of patients, although differences in outcome still exist (59).

Potentially statistical approaches can increase the feasibility of trials in rare diseases. In an early phase, *n* = 1 trials can be used to explore causality. Platform trials, often with a long duration, are commonly used in oncology. The major advantage is the ability to evaluate multiple interventions and the possibility to drop treatment arms and add new ones (60). But also new statistical techniques are being developed for controlled trials. For instance, one could decrease the number of included patients needed to achieve statistical significance by adding real-world controls to a trial. These real-world controls consist of patients who were not included in the trial due to logistic and organizational issues or whose parents chose not to participate in the trial. Considering the VICI trial, more than 425 of the 619 CDH patients who were treated in the VICI trial centers were not included in the trial. These real-world controls would be highly comparable to the VICI trial patients, as they share the same treatment period and the same treatment guideline (i.e., the CDH EURO consortium guidelines), and they were treated in the same centers. Most of the patients not participating were initially treated with conventional mechanical ventilation, because this was standard of care at the time. When combining data from the VICI trial with the observational data from the real-world controls, one needs to account for potential differences in baseline patient characteristics and other biases that may arise from the inclusion of nonrandomized data. In the TOTAL trials, for instance, the outcome in the nonparticipating patients differs from that of the participating patients (5, 6). For these issues, different statistical techniques such as dynamic borrowing can be used (61). This approach would lead to revised estimates of the treatment effect of ventilation mode on the primary endpoint with greater statistical power and precision. Using real-world controls could substantially increase the feasibility of RCTs in a rare patient population. To our knowledge, however, it has not been used in clinical research.

One may also need fewer patients by using a more sensitive primary endpoint than a dichotomous or cross-sectional endpoint. One could incorporate repeated measurements or have a more informative scale (e.g., ordinal or continuous), as it increases the statistical power. For instance, CLD was defined in the VICI trial as the need for any respiratory support on day 28. This definition disregards the amount and the duration of respiratory support. Several additional measurements collected from VICI trial patients could be used to define more informative endpoints. This can include continuous variables such as the degree of oxygen support required, ordinal endpoints such as the level of ventilation support, and derived endpoints such as time to discharge or time to the reduction of ventilatory support. Based on these informative endpoints, multiple hypothesis tests with improved statistical properties compared to the original primary analysis can be applied. One can test each endpoint separately but also combine the endpoints in a single composite endpoint, for instance, by defining a score that incorporates information from the different endpoints and accounts for mortality. Specific statistical approaches to account for multiplicity for testing of multiple, repeatedly measured endpoints will be needed, for instance, the multiple marginal generalized estimating equation (GEE) model method (62). This method can incorporate endpoints on different scales (e.g., death and oxygen support), record measurements at a single time point only, and perform repeated measurements while taking the correlation between endpoints into account to maximize the power of statistical tests. Consultation with biostatisticians at an early stage of trial design is increasingly important to prevent frustration and loss of contributing centers by conducting a classical RCT, especially as newer statistical approaches are on the horizon. The equations should be inserted in editable format from the equation editor.

## Conclusion

So far, only three multicenter clinical trials have been shown to be clinically possible, i.e., two prenatal and one postnatal trials, all with a ventilatory endpoint (2, 5, 6). These trials were very difficult to conduct. New RCTs are recruiting, and those focus on the reduction in PH, a major contributor to mortality in CDH patients. Our experience learns that performing an RCT in CDH patients is challenging. One should timely perform the trial when there is still equipoise. It is essential to select the correct intervention and dose, select the appropriate population of CDH patients, and define a relevant endpoint that also allows a realistic duration and sample size. Also, new statistical approaches might increase the feasibility of RCTs in patients with CDH. But above all, awareness of policymakers for the relevance of investigator-initiated trials should be increased. Possibly European Reference Networks, such as ERNICA, can have a role in improving the climate for these trials. After the implementation of the new European Clinical Trial Regulation, regulators should timely evaluate the effects on investigator-initiated trials, especially in rare diseases such as CDH.

## Author Contributions

SC-dO drafted the initial manuscript and reviewed and revised the manuscript. JD, LS, AG, and DT critically revised the manuscript. All authors contributed to the article and approved the submitted version.

## Funding

SC-dO and DT were supported by a Grant from the Sophia Children's Hospital Foundation (SSWO 17-29) and CDH UK (16EM01).

## Conflict of Interest

The authors declare that the research was conducted in the absence of any commercial or financial relationships that could be construed as a potential conflict of interest.

## Publisher's Note

All claims expressed in this article are solely those of the authors and do not necessarily represent those of their affiliated organizations, or those of the publisher, the editors and the reviewers. Any product that may be evaluated in this article, or claim that may be made by its manufacturer, is not guaranteed or endorsed by the publisher.
